# Identification of a Major Dimorphic Region in the Functionally Critical N-Terminal ID1 Domain of VAR2CSA

**DOI:** 10.1371/journal.pone.0137695

**Published:** 2015-09-22

**Authors:** Justin Doritchamou, Audrey Sabbagh, Jakob S. Jespersen, Emmanuelle Renard, Ali Salanti, Morten A. Nielsen, Philippe Deloron, Nicaise Tuikue Ndam

**Affiliations:** 1 PRES Sorbonne Paris Cité, Université Paris Descartes, Paris, France; 2 UMR216 - MERIT, Institut de Recherche pour le Développement, Paris, France; 3 Centre for Medical Parasitology, University of Copenhagen, Copenhagen, Denmark; Bernhard Nocht Institute for Tropical Medicine, GERMANY

## Abstract

The VAR2CSA protein of *Plasmodium falciparum* is transported to and expressed on the infected erythrocyte surface where it plays a key role in placental malaria (PM). It is the current leading candidate for a vaccine to prevent PM. However, the antigenic polymorphism integral to VAR2CSA poses a challenge for vaccine development. Based on detailed analysis of polymorphisms in the sequence of its ligand-binding N-terminal region, currently the main focus for vaccine development, we assessed *var2csa* from parasite isolates infecting pregnant women. The results reveal for the first time the presence of a major dimorphic region in the functionally critical N-terminal ID1 domain. Parasite isolates expressing VAR2CSA with particular motifs present within this domain are associated with gravidity- and parasite density-related effects. These observations are of particular interest in guiding efforts with respect to optimization of the VAR2CSA-based vaccines currently under development.

## Introduction

Placental malaria (PM) is an important cause of maternal anemia, stillbirth and fetal growth alteration, leading to low birth weight (LBW) babies [1–3]. Furthermore, PM may have long-term consequences for the newborn, LBW representing a major risk factor for infant morbidity and mortality in Africa [4]. The typical feature of PM is the sequestration of *Plasmodium falciparum*-infected erythrocytes (IE) that adhere to chondroitin sulphate A (CSA) in the placental intervillous spaces. VAR2CSA, a member of the *P*. *falciparum* Erythrocyte Membrane Protein 1 (PfEMP1) protein family, plays a crucial role in the binding of IE to CSA [5–7]. Expression of VAR2CSA on the IE surface is the key factor for IE accumulation in the placenta [8]. Several natural immune mechanisms including opsonic phagocytosis [9,10], complement activation [11,12] and agglutination [13,14] against placental isolates have been reported as components of acquired immunity to *P*. *falciparum* infection during pregnancy. Cohort studies clearly suggested that a major mechanism of naturally-acquired protection against PM involves antibody-mediated inhibition of IE binding to CSA [15–18], raising hope of developing a vaccine to prevent PM [18].

VAR2CSA is currently considered as the prime candidate for vaccine development [19] because: (i) VAR2CSA-expressing parasites are the primary cause of PM, (ii) anti-VAR2CSA antibody levels increase with gravidity, as do the levels of antibodies that inhibit IE binding to CSA, (iii) women who have been exposed to PM and having acquired VAR2CSA-specific antibodies give birth to higher weight babies [6,20], and (iv) anti-adhesion antibodies can be induced via immunization in laboratory-animals with VAR2CSA recombinant proteins [21,22].

VAR2CSA is a large trans-membrane polymorphic protein (~350 kDa) consisting of six duffy-binding like (DBL) domains [23]. Recent studies have identified the minimal determining portion of the N-terminal region of VAR2CSA that retains the major amino acids residues targeted by anti-adhesion antibodies as well as the interaction site with CSA [21,24,25]. Although *var2csa* shows a relatively high degree of sequence homology among parasite strains, inter-clonal sequence variations remains high [26,27]. The diversity of this gene in the parasite population is ~ 500-fold higher than a random set of 200 typical *P*. *falciparum* genes [26]. This high level of diversity is a crucial challenge for the development of an effective vaccine. The IE binding inhibitory capacity of antibodies to a given VAR2CSA antigenic construct (FCR3- or 3D7-variant) differed between isolates, being high against counterpart isolates, and absent or weak in some other [28]. Such variant-specific inhibition was previously reported by antibodies induced by the full-length VAR2CSA protein [29]. Placental infection may persist in the presence of high plasma levels of VAR2CSA-specific antibodies [30], highlighting the need for a thorough analysis of the antigenic diversity in the N-terminal part of VAR2CSA to guide optimal vaccine development. Most studies focused on partial gene fragments, often investigating laboratory strains [27,31–35], but few have explored the NTS-DBL1X-ID1-DBL2X sequence polymorphism of VAR2CSA in a global collection of *P*. *falciparum* isolates from different geographical origins [26,31,35]. In the current study, we analyzed polymorphisms in the N-terminal fragment of VAR2CSA expressed by *P*. *falciparum* isolates from Beninese pregnant women, and investigated the relationships between these polymorphisms and a set of biological and clinical parameters.

## Results

### Clinical and parasitological data

Parasite isolates from 46 pregnant women were used. Of these, 14 (30%) were from primigravidae. The women’s gravidity rank ranged from one to eight with a median of 2.00 (interquartile range [IQR], 1–3.25), and mean gestational age (GA) at blood collection was 21.9 ± 10.0 (mean ± SD) weeks. Mean multiplicity of infection (MOI) was 3.01 (range, 1–6). Median parasite density was 4224.5 (IQR, 624.2–30249.3) parasites/μl, ranging from 50 to 224,000. Median *in vitro* binding density of parasite isolates to chondroitin sulphate proteoglycan (CSPG) was 63 (IQR, 2–161) parasites/mm^2^.

### Genetic diversity and polymorphism of NTS-ID2a in isolates

**Fig 1:**
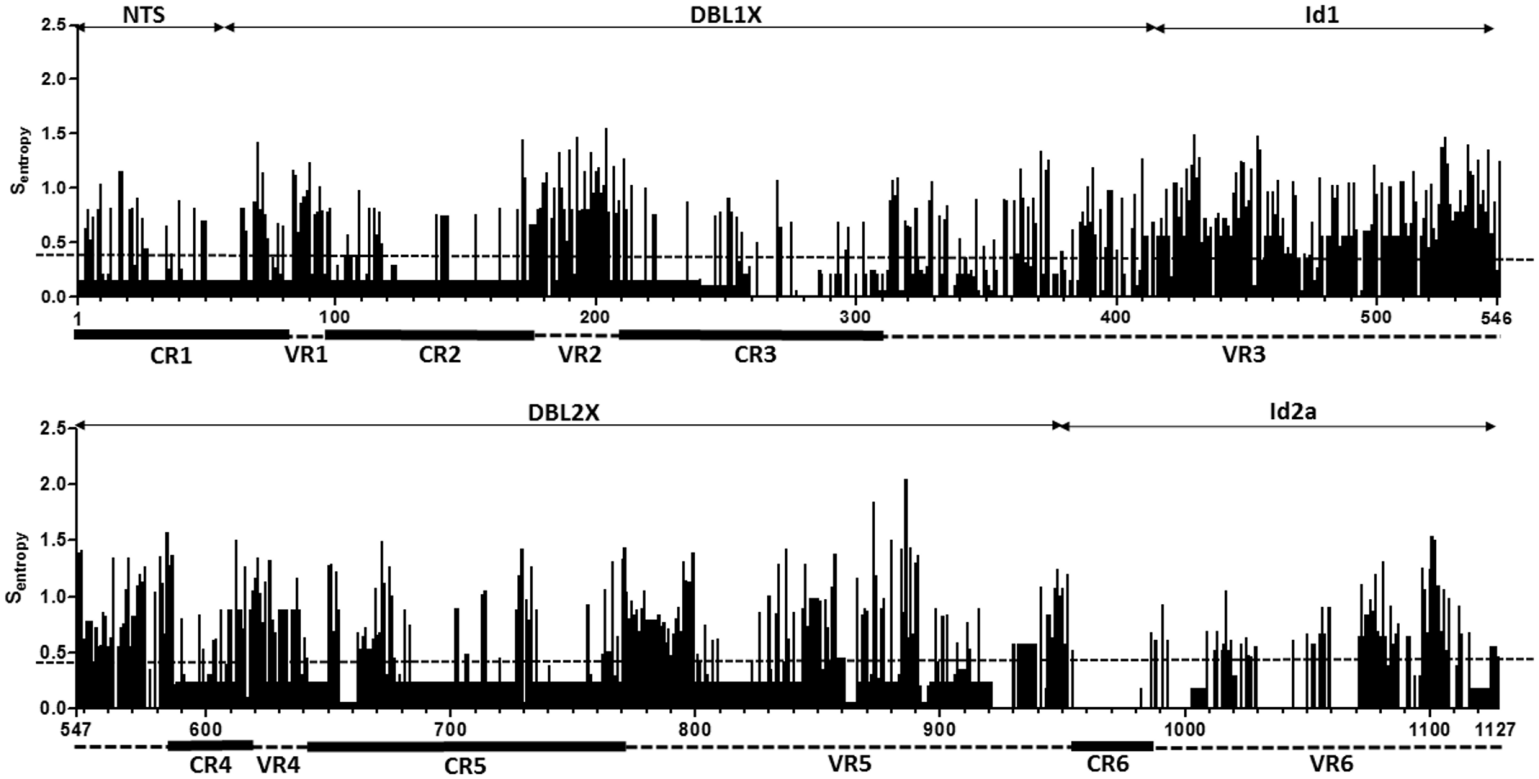
Overview of amino acid sequence variability among NTS-ID2a fragments of VAR2CSA. Shannon entropy values calculated on the multiple sequence alignment of 90 NTS-ID2a amino acid sequences transcribed by parasites collected from pregnant women, were computed. VAR2CSA domains structure and regions covered by sequencing (NTS: N-terminal segment; DBL, Duffy-binding like; ID: inter-domain region) are indicated. Dotted horizontal lines indicate median of all entropy values (H = 0.39). Black-colored boxes (under graphs) delimit conserved regions (CR1-6), while dotted lines indicate variable regions (VR1-6).

The 46 isolates generated 398 NTS-ID2a nucleotide sequences of which 113 were distinct at the nucleotide level. Among these sequences, 90 (80%) were distinct at the amino acid level with an average of two distinct haplotypes per isolate (range, 1–4). Six sequences represented partial coverage of the NTS-ID2a segment. In each isolate, the most frequent haplotype sequence accounted for a mean of 84% of all sequences. The number of distinct haplotypes detected in an isolate was not related to any clinical or parasitological parameter (all *P*-values > 0.05). DNA analysis within the sequenced part of *var2csa* showed that six regions were relatively conserved (CR1–6) separated by six variable regions (VR1–6) (Fig 1, S1 Table). NTS-ID2a polymorphism was analyzed to detect the influence of diversifying or balancing selection by calculating pairwise nucleotide diversity (π) and Tajima’s D value to reveal selection hotspots (S1 Fig). The average pairwise nucleotide diversity observed was 0.12 for the full-length segment of NTS-ID2a. (π values are 0.07 for NTS, 0.10 for DBL1X, 0.19 for ID1, 0.10 for DBL2X, and 0.16 for ID2a). Tajima’s D for the entire NTS-ID2a segment was 1.03 (0.5 for NTS, 0.8 for DBL1X, 1.0 for ID1, 0.15 for DBL2X, and -1.7 for ID2a). This analysis was also performed across the whole nucleotide sequence alignment. Diversity in the NTS-ID2a fragment was primarily located in the variable VR3 region. No significant polymorphism was observed in combination of segments corresponding to CR1-VR1-CR2-VR2-CR3 and CR4-VR4-CR5-VR5-CR6-VR6. Only the VR3 region showed sub-regions with high degree of pairwise nucleotide diversity, and is likely to be subjected to selective pressure (Fig 1 and S1 Fig). VR3 corresponds to the ID1 region of VAR2CSA, suggesting that the diversity of this region has been shaped by balancing selection. Variation of NTS-ID2a amino acid sequences was measured by the variability at each residual position quantified by S_entropy_ score (Fig 1). The median score was 0.39 and regions with S_entropy_ score above the median were defined as highly variable regions. Although the NTS-ID2a sequence segment was not highly variable (64% of sequences similarity), it contains variable sub-units including ID1 (54.8% of similarity).

### Dimorphic sequence motifs (DSM) in the N-terminal segment of VAR2CSA

**Fig 2:**
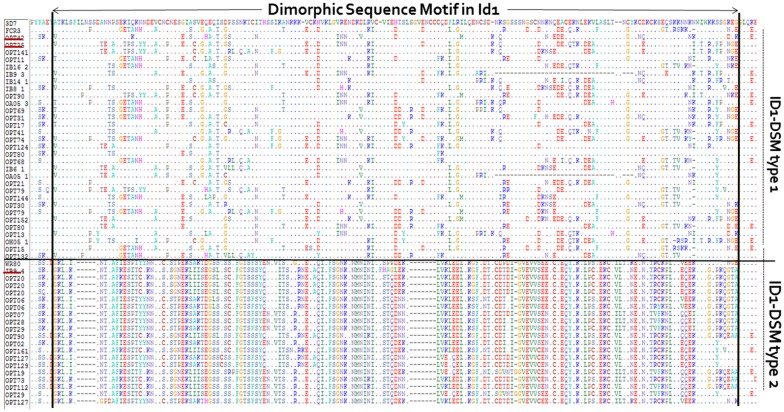
Dimorphic sequence motif in the ID1 domain of VAR2CSA. A window of the multiple sequence alignment, covering ∼198 amino acids and including 3D7, FCR3 and WR80 VAR2CSA sequences (underlined in red) was illustrated. 3D7 is used as reference for the alignment and for each sequence below, dot at a residue position represents identical amino acid to 3D7 sequence. The dimorphic sequence motif (DSM) is indicated. Selected sequences sharing the similar ID1-DSM type are separated by a solid horizontal line. 3D7 and FCR3 strains exhibited the ID1-DSM type 1 and WR80 is part of ID1-DSM type 2 sequences.

Positional amino acid conservation analysis was performed to identify critical motifs and important residues in the aligned NTS-ID2a region. Three NTS-ID2a sequences retrieved from GenBank (Accession numbers: AY372123 for FCR3; EF614228 for WR80 and AAN36095 for 3D7) were included in the analysis for comparison purposes. The analysis showed several conserved sequence patterns, interspersed with variable sequence blocks. A segment in ID1 clearly showed a dimorphic amino acid sequence between positions 416 and 596 (Fig 2), and segregated sequences into two distinct groups. First variant or ID1-DSM type 1 (∼ 172 amino acids) was found in 68 (76%) sequences from isolates as well as in FCR3 and 3D7 lines (*Cluster 1*). Twenty-two (24%) field isolates exhibited the second variant of the dimorphism or ID1-DSM type 2 (∼162 amino acids), also present in the WR80 line (*Cluster 2*).

The DSM described in the DBL2X domain of VAR2CSA [27] was also present in this independent set of sequences. The FCR3-type variant of DSM in DBL2X was present in 44% of sequences, and the 3D7-type variant in 56% of sequences from isolates.

**Fig 3:**
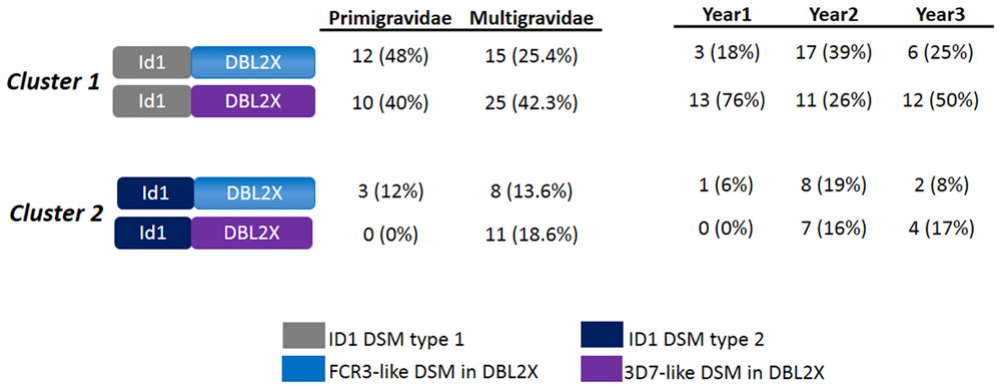
Gravidity and haplotype combination of ID1 and DBL2X DSM. Relationship between the gravidity of women, year of parasite collection and the combination of haplotypes defined by DSM in ID1 and DBL2X fragments is shown. Number of sequences sharing ID1-DSM type 1 (*Cluster 1*) or ID1-DSM type 2 (*Cluster 2*) and FCR3- or 3D7-like DSM in DBL2X were presented. Prevalence of each defined haplotype in primigravidae and multigravidae is indicated. Parasites were collected from three studies conducted between 2008 and 2010 (Year1 = 8 isolates), in 2012 (Year2 = 25 isolates) and 2013 (Year3 = 13 isolates). Prevalence of the haplotypes is indicated for each year of collection.

Haplotype combination analysis of DSM in ID1-DBL2X fragment was conducted according to gravidity and the years of parasite collection (Fig 3). Importantly, sequences from *Cluster 2* that carried the 3D7-like DSM in DBL2X were exclusively detected in isolates from multigravidae but not in the Year1 of collection where the majority of the isolates (6/8) were collected from multigravidae. An increasing trend in the prevalence of *Cluster 2* sequences and especially this particular haplotype along the years was observed.

### Divergence of NTS-ID2a sequences is dictated primarily by the dimorphic signature in ID1

**Fig 4:**
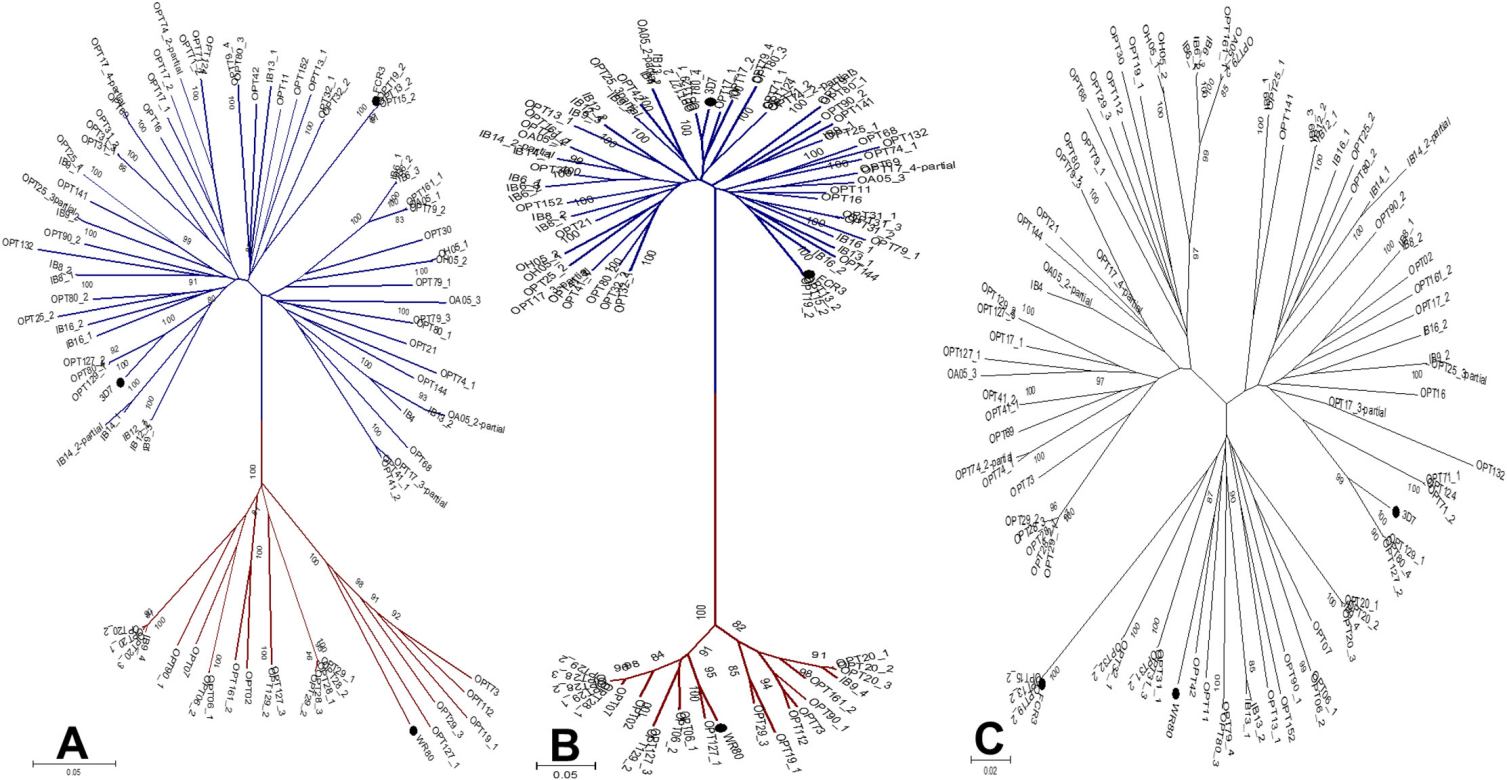
Phylogenetic relationships among sequences of NTS-ID2a fragments of VAR2CSA. Phylogenetic relationship between the sequences was illustrated by Neighbor joining trees computed in MEGA6. Phylogenetic tree corresponding to NTS-ID2a (**A**), ID1 (**B**) and after excision of the ID1-DSM region in NTS-ID2a (**C**) alignments were presented. Branches of the two major DSM variants of ID1 were highlighted in separate colors (blue for *cluster 1*’s taxa and red for taxa members of *cluster 2*). FCR3, 3D7 and WR80 used for comparison purpose were indicated with a black dot. The bootstrap values (>80) is shown at the main bifurcation.

Phylogenetic sequences analysis generated trees with substantially different branch lengths (Fig 4). The NTS-ID2a sequences clustered into two phylogenetic subgroups (bootstrap value 100) (Fig 4A). Dichotomy carried by the DBL2 DSM was rather observed in one of the two major phylogenetic groups [27], as if it was less important to be muffled by that imposed by ID1. Distinct sequences identified in one isolate may cluster into different phylogenetic subgroups. Phylogenetic trees corresponding to the NTS-DBL2X sub-units (DBL1X and DBL2X) (S2 Fig) confirmed that the dichotomization is primarily driven by variations in the ID1 segment (Fig 4B and 4C).

### Cysteine composition of ID1 dimorphic sequence

The number of cysteine residues was assessed in the ID1 DSM from both sequence clusters to assess differences that might influence the conformational/functional properties of VAR2CSA. Most *Cluster 1* sequences contained either 12 (29%) or 13 (31%) cysteine residues in the ID1 DSM, whereas a minority contained 8 to 10 cysteine residues (S3 Fig), and no sequence contained 15 cysteine residues. In *Cluster 2*, 41% sequences contained 13 cysteine residues whereas 11, 12, and 15 cysteine residues were found in 9%, 18%, and 14% of sequences, respectively. No sequences with 8 to 10 cysteine residues were found in *Cluster 2*. No significant association between the number of cysteine residues in the ID1 DSM and parasitological parameters including CSPG binding ability was observed.

### Association of ID1 DSM with clinical and parasitological parameters

We evaluated whether the two ID1 sequence clusters were associated with different clinical or parasitological features (Table 1). Isolates from *Cluster 2* were associated with higher parasitemia than isolates from *Cluster 1* (59,833 vs 18,794 parasites /μl, *P*-value = 0.006), and were less found in primigravidae (14.3% vs 39.3%, *P*-value = 0.03). Although higher mean parasitemia was observed in multigravidae infected with parasites belonging to cluster 2, no difference was found in the parasitemia according to the gravidity of women (Mann-Whitney test, *P* = 0.99).

**Table 1:** Comparison of phenotypic data between the two clusters of sequences defined according to the ID1 DSM.

	*Cluster 1*	*Cluster 2*	*P*-value^†^
**Parasite density (parasites/μl)** *	18794 ± 38310.4	59832.7 ± 68050.6	0.006
**Gestational Age (weeks)** *	22.5 ± 10.8	21.2 ± 8.6	0.29
**Proportion of primigravidae**	39.3%	14.3%	0.03
**CSPG binding density (parasites/mm** ^**2**^ **)** *	128.4 ± 158.6	140.4 ± 204.2	0.68
**Proportion of high CSPG binders**	52.1%	41.2%	0.57

*Data expressed as mean ± standard deviation.

^†^ Mann-Whitney test

### Genotype-phenotype correlations at the residue level

A set of predictions (Z-scores) estimated the strength of the association of each residue type at each variable position of the NTS-DBL2X protein sequence alignment to each clinical and parasitological parameter. Four residues at positions 872, 883, 885 and 887 were associated with parasite ability to bind CSPG (*P* = 0.0002, 0.0001, 0.049 and 0.0009, respectively after Bonferroni correction). High CSPG binding correlated with amino acids N_872_, E_883_, R_885_, and T_887_. Table 2 shows the prevalence of each residue in low-or high- CSPG binding isolates. Although the associated residues are very close to each other, their lack of linkage disequilibrium does not allow identification of true sequence motifs involving several contiguous sites. Several amino acids of NTS-DBL2X sequences were also associated with gravidity (positions 450, 464 and 466, with Bonferroni-corrected *P* = 0.0002, 0.0009, and 0.018, respectively). These positions are located within the ID1 DSM. Visual inspection of the region around these three positions in the multiple alignments of protein sequences revealed distinct motifs between primigravidae and multigravidae. Three motifs spanning amino acids 461 to 468 showed a differential distribution according to gravidity (Table 3). Two of them (NTHSSIKA and NTHSSIKT) were predominantly found in primigravidae (*P* = 0.0008, Fisher's exact test), while the third one (SYENSVTS) was exclusively found in multigravidae (*P* = 0.004; Table 3).

**Table 2:** Association of amino acid residues in the NTS-DBL2X segment with CSPG binding density.

Amino acid residue	Position	Prevalence in CSPG low-binders	Prevalence in CSPG high-binders	*P*-value^a^	OR [95% CI]
**N**	872	8.8%	37.5%	0.008	6.0 [1.4–37.4]
**E**	883	26.5%	78.1%	0.00003	9.5 [2.8–36.3]
**R**	885	5.9%	39.1%	0.021	6.1 [1.1–63.3]
**T**	887	38.2%	81.3%	0.0005	6.8 [2.0–25.9]

^a^Two-tailed Fisher’s exact test.

OR, odds ratio; CI, confidence interval.

**Table 3:** Association of NTS-DBL2X sequence motifs with gravidity of pregnant women.

Positions 461–468	Primigravidae (*n* = 27 sequences)	Multigravidae (*n* = 55 sequences)	*P*-value^a^	OR [95% CI]
**NTHSSIK[A/T]**	13 (48.1%)	7 (12.7%)	0.0008	6.2 [1.9–22.3]
**SYENSVTS**	0 (0%)	14 (25.5%)	0.004	∞ [2.0-∞]

^a^Two-tailed Fisher’s exact test.

OR, odds ratio; CI, confidence interval.

No significant association was found with GA neither as a quantitative variable (number of gestational weeks) nor as a discrete variable. Nonetheless, among the 20 first amino acid residues showing the strongest association with parasitemia, 18 (90%) were located within the ID1 DSM, between positions 416 and 596 (S2 Table). This suggests that the molecular signature carried by the ID1 fragment of VAR2CSA might be important in the physiopathology of malaria in women infected by placental-type parasites.

## Discussion

Several lines of evidence support the N-terminal part of VAR2CSA as the prime target for vaccine development against PM. First, the minimal binding region to placental CSA has been defined in this fragment [25]. Second, antibodies to antigenic constructs from this region of VAR2CSA show greater inhibitory property than antibodies to other regions [21,24,28]. Third, naturally-acquired IgG affinity-purified against this region show anti-adhesion activity [21]. Fourth, high levels of naturally-acquired antibodies to this region are associated with a lower risk of LBW [20]. Overall, these observations support ongoing efforts to develop a VAR2CSA-based vaccine by focusing on its N-terminal fragment (Placental Malaria Vaccine and Priming Malaria Vaccine ongoing projects). VAR2CSA polymorphisms expressed by field isolates are a major challenge to overcome to design an effective vaccine. *P*. *falciparum* parasites modulate several mechanisms to evade the host immune response. These mechanisms include sequence polymorphism, antigen redundancy, clonal antigenic variation, and modulation of cell-mediated immunity [36]. Sequence polymorphism analysis at the population level is an effective tool to explore sites experiencing selection. Insight analysis of sequence variability within the fragment of interest will help to define major variants of VAR2CSA present in field isolates, and to understand the evolution of diversity allowing the parasite to escape vaccine [36].

We investigated the variability at nucleotide and protein levels using NTS-ID2a sequences generated from *P*. *falciparum* parasites infecting pregnant women in Benin. *Var2csa*, is more conserved than other *var* gene members, but its sequences demonstrate variability in defined areas [31]. The sequences analyzed in this study showed a high level of diversity, even within subunit truncations (DBL1, ID1, and DBL2). The pairwise nucleotide diversity of NTS-ID2a and subunits is higher than that of DBL2 and DBL3 domains of VAR2CSA [33,34]. Differences in the geographic origin of parasites used in this study and those analyzed in these previous studies conducted in East and South-Eastern Africa may account for the difference in the level of sequences polymorphism. The high degree of polymorphism we observed in our dataset suggests an important inter-parasite diversity among isolates from Benin. Evidence of this polymorphism within NTS-ID2a is supported by evolutionary tests detecting sites under selection pressure, indicating that diversifying and balancing selection are acting on the NTS-ID2a region. Our observations complement earlier observations that the N-terminal region of VAR2CSA is under diversifying selection [26,31]. As positive selection promotes escape mutations, this finding suggests that balancing selection within this region promotes polymorphism [26] and delayed acquisition of strain-transcendent protective immunity [37].

Our results show that the hyper-variable region within the NTS-ID2a fragment covers the ID1 region. Analysis of amino acids polymorphism highlighted a dimorphic sequence within ID1. This ∼167 amino acids sequence motif is the largest dimorphic region described in PfEMP1. Sander *et al*. reported a 26 amino acids DSM in DBL2X, allowing to derive sequences from isolates in two distinct phylogenetic groups, one group containing FCR3-like variants and the other sequences sharing the 3D7-like DSM [27]. Involvement of this DBL2X dimorphism in the functionality of the protein remains unproven. Although we also observed the DBL2X DSM (S2 Fig), the phylogenetic analysis showed a different clustering pattern. NTS-ID2a sequences clustered in two major groups mainly driven by the ID1 dimorphism. In this new clustering pattern, FCR3- and 3D7-like variants, as defined by the DBL2X DSM, are combined in the same large cluster, and a second cluster contains rarely described variants of *P*. *falciparum* such as WR80 (originating from South-East Asia) [31], not documented in Africa so far. The high proportion (25%) of WR80-like variants and the increase in the prevalence of this variant along the years of collection may indicate an emergence of such variants among VAR2CSA expressing parasites. However, evidence of new clonal expansion of this variant need to be verified in further studies.

The ID1 region is part of the putative residues that binds CSA [25], and induces anti-adhesion antibodies against placental-type parasites [24]. Sequence variations in ID1 may modify the functionality of antibodies against antigenic constructs including ID1. The positive association between isolates sharing specific dimorphic signatures in ID1 and high parasite density in pregnant women suggests that these isolates are more virulent and more likely to cause adverse pregnancy outcomes. Parasites carrying the WR80-like ID1 *var2csa* haplotype were more prevalent among multigravidae, where the associated 3D7-like DSM in DBL2X haplotype was exclusively found. The fact that these parasite variants were mostly or exclusively found among multigravidae is an observation that challenges a possible way of VAR2CSA-expressing parasites to escape acquired immunity against PM. This is more plausible that these isolates were associated with high parasite densities in women who generally have a pre-existing anti-VAR2CSA immunity. The high proportion (25%) of WR80-like variants, mainly found in multigravidae might partially explain the fact that no difference has been observed in the parasite density between primigravidae and multigravidae in this study. The sequence motifs NTHSSIKA/T and SYENSVTS defined within the ID1 were associated with gravidity, while the SYENSVTS motif was exclusively detected in parasites from multigravidae. The SYENSVTS motif was typical of VAR2CSA haplotype with a WR80-like DSM in ID1 that was associated with a 3D7-like DSM in the DBL2X. It is possible that parasites expressing these VAR2CSA haplotypes represent a relatively rare population with respect to those whose first-time pregnant women are mainly exposed to. High plasma levels of anti-VAR2CSA antibodies that is characteristic of multigravid women in malaria endemic settings, is associated with protection against placental malaria infections [20]. Preferential (or exclusive) infection of these women by isolates with the WR80-like variant molecular signature might suggest that antibodies acquired against common VAR2CSA variants do not prevent or allow the control of infections with these variants. The binding ability of the studied isolates to CSPG was unrelated to ID1 polymorphisms. One field isolate carrying the WR80 signature that we established in *in vitro* culture had a similar ability to bind CSA as other laboratory-adapted CSA-binding strains (data not shown), suggesting that this polymorphism does not affect the parasite’s ability to bind to CSA per se. These findings have major implications for an optimal design of a VAR2CSA-based vaccine.

Sequence analysis of the DBL2X domain of VAR2CSA highlighted that amino acids N_872_, E_883_, R_885_ and T_887_ were associated with high CSPG binding. Although these positions are very close, no sequence motif was associated with high CSA binding phenotype. Nevertheless, their presence in DBL2X suggests their potential involvement in the CSA binding key residues, and this sequence motif (NxxxxxxxxxxExRxT) might be the canonical sequence of high CSA-binding parasites.

Development of a VAR2CSA-based vaccine faces a major obstacle of substantial antigenic diversity. A multivalent VAR2CSA vaccine candidate able to induce a broad antibody repertoire against the most common and biologically relevant variants may overcome antigenic diversity [36,38]. Such parameters should be considered in the ongoing efforts to develop a promising vaccine.

## Materials and Methods

### Study site and sample collection

Samples were collected from pregnant women during studies conducted between 2008 and 2013 [21,28,39] in health centers of southern Benin where transmission of *P*. *falciparum* malaria is hyper-endemic with an entomological inoculation rate ranging from 35 to 60 infective bites per person and per year [40]. *P*. *falciparum* infection among pregnant women who presented to the health centers for an antenatal visit or for delivery was identified by a rapid diagnostic test (Parascreen) and was confirmed by microscopy. Women were enrolled after obtaining signed informed consent, and venous blood was collected. All studies were approved by the Ethics Committee of the Faculty of Health Sciences, University of Abomey-Calavi (Benin).

Blood samples were centrifuged and 200μL of erythrocyte pellets were frozen either at -20°C for DNA extraction or in 9 volumes of Trizol at -80°C for subsequent total RNA extraction. The remaining fraction of the pellet was immediately cultured *in vitro* to obtain late-stage asexual parasite forms.

### In vitro binding capacity of infected erythrocytes

The binding level of IE to CSPG was assessed on a static assay, as described [39]. Briefly, late stage—infected IE enriched by filtration over a magnetic column (MACS) were blocked in BSA/RPMI buffered solution and allowed to bind to CSPG (coated as spots in a 100 × 15mm Falcon 351029 Petri dish) for 15 minutes at room temperature. Non-adhering cells were removed by an automated washing system. Adhering IE were fixed with 1.5% glutaraldehyde in PBS, Giemsa-stained, and microscopically quantified under oil immersion at x100 magnification.

### Genomic DNA, RNA extraction and cDNA synthesis

Genomic DNA (gDNA) was extracted from erythrocyte pellets using *QIAamp DNA Blood extraction kit*, as recommended by the manufacturer (Qiagen). *Msp1* and *msp2* genes were amplified by nested PCR, as described [41]. The multiplicity of infection (MOI) was determined for each sample.

Total RNA was extracted from erythrocyte pellets preserved in Trizol as recommended by the manufacturer (Invitrogen). RNAs were treated with DNase I (Invitrogen) to remove possible contamination of gDNA, as described [42]. Complementary DNAs (cDNA) were synthesized by reverse transcription of DNA-free RNA using Thermoscript (Invitrogen) with random hexamer primers for 1 hour at 50°C, as recommended by the manufacturer.

### Amplification, cloning and sequencing

The region of the *var2csa* gene (*PFL0030c*) covering the NTS-ID2a fragment, nucleotide positions 1–3100 (3100 bp) was amplified from the cDNA of pregnant women isolates, using the high fidelity Fusion Taq Polymerase (New England Biolabs). Primers Fw 5’-ATGGATAAATCAAGTATTGCT-3’ and Rv 5’-GAACAGTGGAACAAAGAAATAC-3’ were used under the cycling conditions: 94°C for 1 min, followed by 35 cycles of 94°C for 30 s, 50°C for 30 s and 68°C for 3 min 40 s, with a final extension at 68°C for 10 min. The PCR products were subjected to electrophoresis and purified using the "PCR clean-up, Gel extraction" kit (Macherey-Nagel). Amplicons were ligated into pCR™-Blunt II-TOPO plasmid (Invitrogen) and transformed into One Shot competent bacteria using the TOPO cloning kit—Zero Blunt (Invitrogen), as recommended by the manufacturer. All colonies were analyzed by PCR using the TEMPase Hot Start DNA polymerase (Ampliqon) and the flanking universal primers M13F / M13R. Ten clones were selected per sample. DNA was sequenced from selected clones at GATC biotech (Cologne, Germany).

### Sequence Analysis

The different pieces of generated sequences were assembled using DNA Dragon (Sequentix) software to a full sequence of NTS-ID2a. The nucleotide sequences were analyzed using BioEdit 7.1 to sort those belonging to the same variants according to their similarity. Sequences differing by 10 or more nucleotides (99%) in pairwise comparisons were considered as true unique sequences. Tajima’s D test as implemented in MEGA 6 [43] and DnaSP [44], and pairwise nucleotide diversity (π) were used to analyze sequence diversity. Elevated π and positive values of D would confer a balancing selection of nucleotide sites that would be maintained at intermediate frequencies.

Sequences were translated into amino acids and the multiple alignment of amino acid sequences was generated using MAFFT Version 7 (http://mafft.cbrc.jp/alignment/server/) and manually corrected. The Shannon entropy (S_entropy_), which represents a robust approach to the measurement of the sequence variation [45], was used to assess the degree of amino acids variation at each position according to the multiple alignment of protein sequences. The entropy score at each position of the alignment was calculated using BioEdit [46] and plotted using GraphPad Prism version 5.00 (GraphPad Software, San Diego California USA, www.graphpad.com). All sequences have been submitted to GenBank with accession numbers KT359638—KT359727. Subunits within NTS-ID2a were analyzed according to limits previously described by Andersen et al. [47]. Phylogenetic trees were generated using the Neighbor-Joining algorithm implemented in MEGA 6, with the p-distance method as substitution model. Branch supports were estimated from 1,000 bootstrap replicates.

### Genotype-phenotype mapping

Association of specific sequence motifs with clinical or parasitological parameters was investigated by a genotype-phenotype correlation analysis based on the protein multiple sequence alignment. The SigniSite 2.1 server [48] was used for quantitative phenotypes (GA [weeks], peripheral blood parasite density [parasites/μL of blood], and CSPG-binding density [parasites/mm^2^]). The SPEER-Server [49] was used for binary phenotypes (gravidity [primigravidae *vs* multigravidae], CSPG-binding status [low *vs* high binding parasites, considering the median (63 parasites/mm^2^) as a threshold value] and GA using a cut-off at 16 weeks of gestation (median value) and by grouping into trimester of pregnancy [1^st^ trimester: GA ≤ 13 weeks; 2^nd^ trimester: 13 weeks < GA ≤ 26 weeks and 3^rd^ trimester: GA > 26 weeks]). The genotype-phenotype mapping analysis was carried out only for the NTS-DBL2X segment since the number of sequences available up to ID2a was too low for meaningful analysis. Parameter values associated with a given parasite isolate were assigned to all NTS-DBL2X sequences identified in that particular isolate. When phenotype data were missing for an isolate, the corresponding sequences were excluded from analysis. Invariant positions (ie, with a single amino acid type) and positions with gaps in > 20% sequences were excluded. In both analyses, the normal distributed Z-scores were converted into p-values by standard method. An amino acid residue was considered associated with a phenotype if the *P*-value for the specific residue was smaller than or equal to α = 0.05 after Bonferroni correction for multiple testing.

### Statistical analysis

Possible associations of the sequence variation on clinical and parasitological phenotypes were tested with each of the two clusters of sequences. Fisher's exact tests and Mann-Whitney tests were used to test association with binary (gravidity, CSPG-binding status) and continuous (gestational age, parasite density and CSPG-binding density) phenotypes, respectively, using Stata version 11 for Windows (Stata Corp, College Station, TX, USA).

## Supporting Information

S1 Fig Tajima’D and pairwise nucleotide diversity for NTS-ID2a nucleotide sequence alignment. Tajima’s *D* scores and π values were computed across the whole NTS-ID2a alignment by using a sliding window approach with a window length of 100bp and a step size of 25bp. Subunits, variables and conserved regions within NTS-ID2a are indicated.
(TIF)

S2 Fig Phylogenetic trees of DBL1X and DBL2X domains of VAR2CSA. Trees corresponding to VAR2CSA DBL1X and DBL2X sequences from Beninese pregnant women’s parasites were computed in MEGA6. FCR3, 3D7 and WR80 were indicated with a black dot.
(TIF)

S3 Fig Cysteine composition of ID1-DSM. Number of cysteine residues within the ID1-DSM region of sequences from both DSM variants was plotted. Prevalence of sequences sharing the same number of cysteine residues are indicated for each cluster of sequences.
(TIF)

S1 Table Description of conserved nucleotides regions in NTS-ID2a fragment of VAR2CSA. (DOCX)

S2 Table List of the first 20 amino acid residues within NTS-DBL2X showing the strongest association with host parasitaemia. (DOCX)
