# Supplementary figures and images for: Identification of a Major Dimorphic Region in the Functionally Critical N-Terminal ID1 Domain of VAR2CSA
Source: PLoS One. 2015 Sep 22;10(9):e0137695. doi: 10.1371/journal.pone.0137695 (PMC4579133; doi:10.1371/journal.pone.0137695)

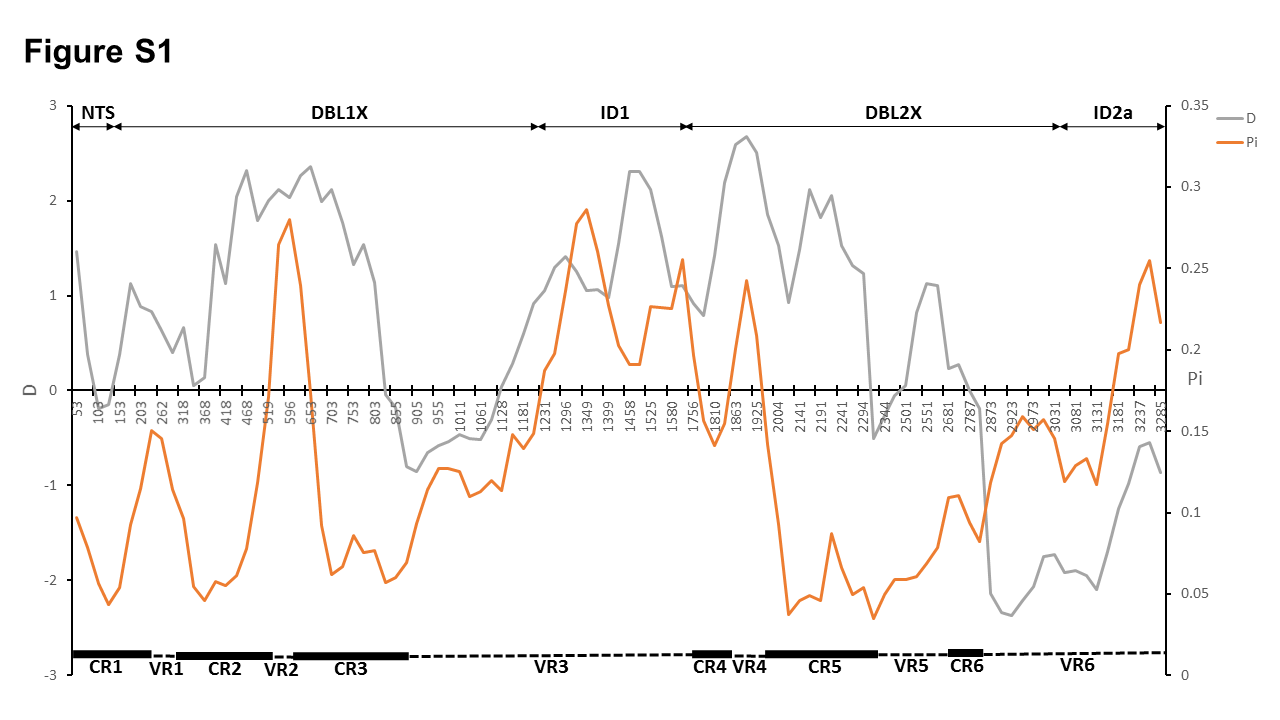

Supplement: S1 Fig — Tajima’s D scores and π values were computed across the whole NTS-ID2a alignment by using a sliding window approach with a window length of 100bp and a step size of 25bp. Subunits, variables and conserved regions within NTS-ID2a are indicated. (TIF) [file pone.0137695.s001.TIF]

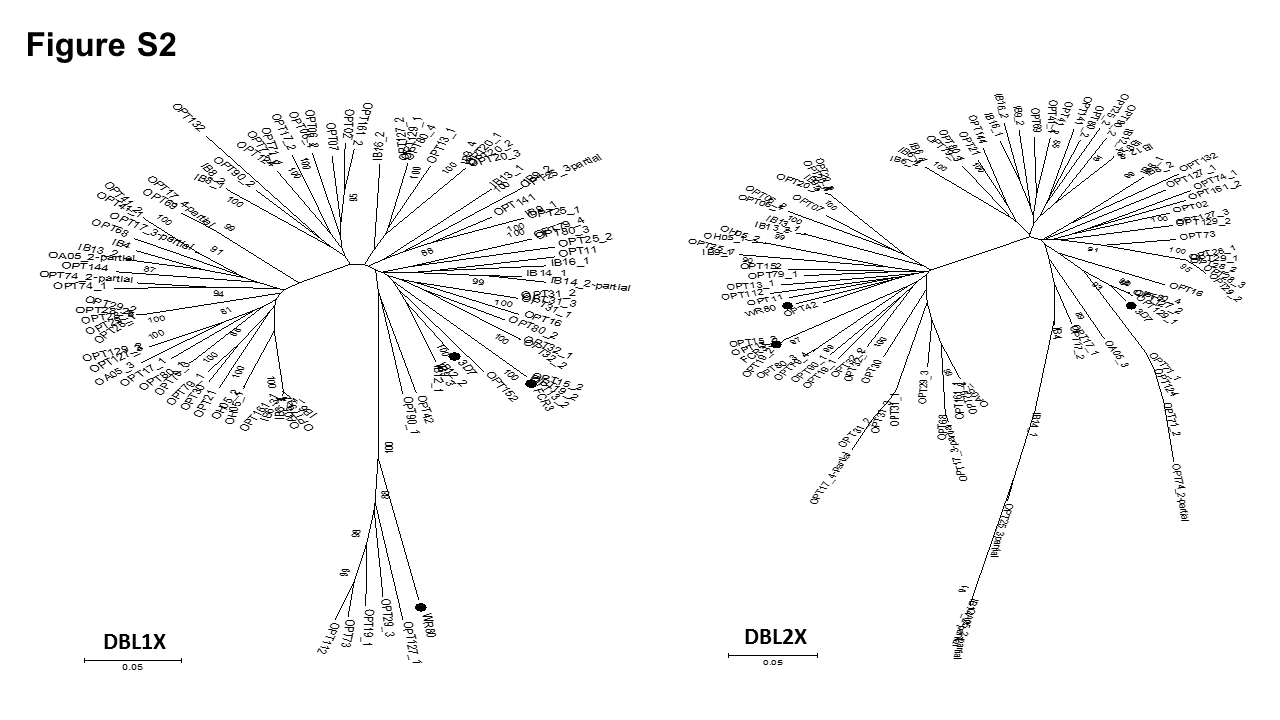

Supplement: S2 Fig — Trees corresponding to VAR2CSA DBL1X and DBL2X sequences from Beninese pregnant women’s parasites were computed in MEGA6. FCR3, 3D7 and WR80 were indicated with a black dot. (TIF) [file pone.0137695.s002.TIF]

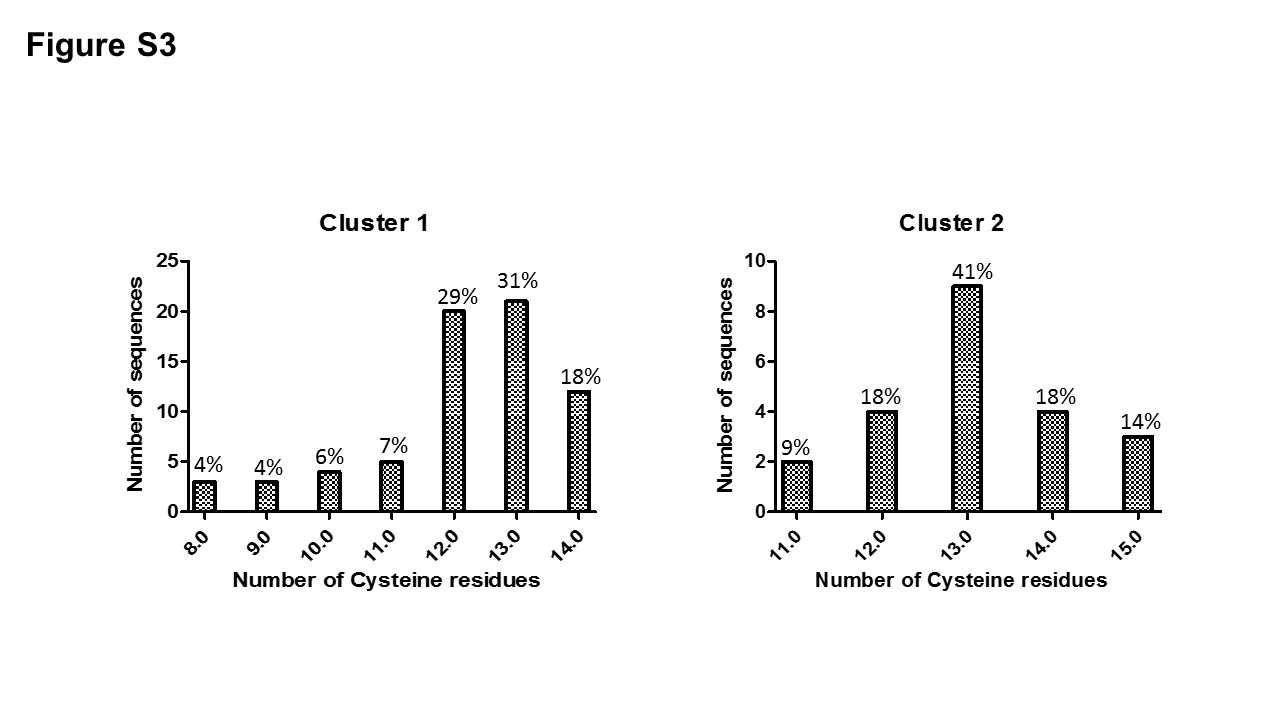

Supplement: S3 Fig — Number of cysteine residues within the ID1-DSM region of sequences from both DSM variants was plotted. Prevalence of sequences sharing the same number of cysteine residues are indicated for each cluster of sequences. (TIF) [file pone.0137695.s003.TIF]
