# Supplementary material for: Identification of a Major Dimorphic Region in the Functionally Critical N-Terminal ID1 Domain of VAR2CSA
Source: PLoS One. 2015 Sep 22;10(9):e0137695. doi: 10.1371/journal.pone.0137695 (PMC4579133; doi:10.1371/journal.pone.0137695)
Supplement: S1 Table — (DOCX) [file pone.0137695.s004.docx]

**Table S1: Description of conserved nucleotides regions in NTS-ID2a fragment of VAR2CSA**

| DNA conserved Region | Length | Positions | Conservation | Homozygosity | P-value |
| --- | --- | --- | --- | --- | --- |
| CR1 | 248 | 1 - 248 | 0.69 | 0.91 | <0.0001 |
| CR2 | 270 | 273 - 542 | 0.73 | 0.91 | <0.0001 |
| CR3 | 306 | 599 - 904 | 0.70 | 0.91 | <0.0001 |
| CR4 | 88 | 1755 - 1842 | 0.65 | 0.88 | 0.03 |
| CR5 | 364 | 1951 - 2314 | 0.74 | 0.92 | <0.0001 |
| CR6 | 105 | 2665 - 2769 | 0.68 | 0.91 | 0.0042 |
