# Supplementary material for: Identification of a Major Dimorphic Region in the Functionally Critical N-Terminal ID1 Domain of VAR2CSA
Source: PLoS One. 2015 Sep 22;10(9):e0137695. doi: 10.1371/journal.pone.0137695 (PMC4579133; doi:10.1371/journal.pone.0137695)
Supplement: S2 Table — (DOCX) [file pone.0137695.s005.docx]

**Table S2: List of the first 20 amino acid residues within NTS-DBL2X showing the strongest association with host parasitaemia**

| **Position** | **Amino acid residue** | **Sens de l'association with parasitaemia**^†^ | **Z-score**^‡^ | ***P*-value**^§^ |
| --- | --- | --- | --- | --- |
| 713 | A | + | 3.76 | 1.73E-04 |
| 535 | N | - | 3.51 | 4.53E-04 |
| 550 | A | - | 3.38 | 7.14E-04 |
| 541 | C | - | 3.38 | 7.14E-04 |
| 552 | L | - | 3.38 | 7.14E-04 |
| 523 | N | - | 3.38 | 7.14E-04 |
| 530 | N | - | 3.38 | 7.14E-04 |
| 527 | S | - | 3.38 | 7.14E-04 |
| 528 | S | - | 3.38 | 7.14E-04 |
| 529 | S | - | 3.38 | 7.14E-04 |
| 532 | S | - | 3.38 | 7.14E-04 |
| 551 | S | - | 3.38 | 7.14E-04 |
| 558 | Y | - | 3.38 | 7.14E-04 |
| 533 | C | - | 3.32 | 9.00E-04 |
| 531 | G | - | 3.22 | 1.30E-03 |
| 528 | * | + | 3.04 | 2.40E-03 |
| 806 | T | + | 3.04 | 2.41E-03 |
| 421 | A | - | 2.94 | 3.28E-03 |
| 424 | A | + | 2.94 | 3.28E-03 |
| 422 | N | - | 2.94 | 3.28E-03 |

**Footnote:**

* Gap representing a deletion

^†^ Positive or negative association of the amino acid residue with host parasitaemia (denotes Z<0 or Z>0).

^‡^ The absolute value of the Z-score computed by Signisite.

^§^ P-value uncorrected for multiple testing.

The 18 positions located within the ID1 DSM are shaded in grey.
